# The National Mission

**Published:** 1916-09-23

**Authors:** 


### The Hospital

The Workers' Newspaper of Administrative Medicine and Institutional Life, Administration, National Insurance and Health.

No. 1581, Vol. LX. SATURDAY, SEPTEMBER 23, 1916. PRICE ONE PENNY.

### THE NATIONAL MISSION.

As students of the past history and present position of Great Britain, we welcome the effort which is to be made in the month of November through the Churches to unite all sects and sections of the population, to awaken them to a realisation of their spiritual nature, and so to prepare for the reception of our warriors at their home-coming after the war, and for future developments in all directions. It has been the immediate business and purpose of The Hospital to promote everything calculated to improve and sustain the good physical condition of every man and woman amongst us. The war has changed many things for the better, and amongst them it has promoted the substitution of realities for shams.

One great thing which a large number of people in Great Britain have ceased to appreciate is their spiritual nature. Before the war there was great spiritual and mental stagnation amongst all classes, which coincided not only with their acceptance of the money standard in all work, including creative work—art and literature—but with the soul-killing worship of silly fetishes in social life which was evidenced by a multitude of conventions separating off layer upon layer of sub-classes. The physician has exceptional opportunities to observe the mental outlook of varying social grades, and the contempt of, say, Wandsworth for Balham, or of the family with a car for the family on bicycles, is typical of the sort of degeneration we have in mind. It is far more deep-seated and widespread than the broad-minded may possibly believe. "*What have you got in material things?*" is a test that has long overshadowed "*What is your value as a spiritual being, as a child trying to express, within human limitations, the glory, love, and wisdom of its Heavenly Father?*" The sterility of inter-family relations and the tragedy of internal family feuds, due entirely to want of sympathy and understanding based on the life spiritual, is one of the very important things which need prompt removal. Dr. Jowett recently bore testimony from a letter which an intelligent soldier had written from the Front in reply to a question as to the spiritual tendency of the men. The writer said that "isms"—Wesleyanism, Catholicism, and all the rest—"were going rottenly, but that the men were laying hold of God in splendid fashion."

The truth is, unless we are wholesome in spirit we cannot be healthy in body. The movement by the Churches in November to arouse the men and women throughout the nation to the recognition of their spiritual nature, and to lay hold of God as our warriors are doing, has our entire sympathy. We have given a good deal of thought to this matter; we feel that every man and woman, and especially every medical man and all who are intimately associated with the sick, should make it their special business to ask himself where he stands in this matter, and what he can do individually to help forward the movement to awaken people of all classes, as our soldiers have been awakened, since the war, when standing face to face with death at the Front. Our readers are largely identified with the medical profession, and institutional and social life throughout the country. The physician and the Churchman have often been closely associated together in history as they have been associated at the Front during the existing war.

It would be of immense value to the future of the nation and Empire if the serious view of life now taken by our fighting men could be taken by the people at home. We want more life in the spiritual sense, individually and collectively. How this can be brought about is a problem well worthy of the consideration of the best types of men and women in all ranks of society.

With a view to help reflection and to facilitate a study of the problems presented, and of the ways to overcome them, we commence this week a series of articles entitled "Life and Death: Some Human Lessons in War-Time." We propose to publish an article each week up to the beginning of November, when the great Mission for all the Churches is timed to commence. The series, it is hoped, will include articles on "The Nation and the Great War," "The Physician and the Preacher," "The Pleasures of Life," "Riches and Wealth," "Childhood and the Children," "The Psychology of Popularity," "The Roots of True Statesmanship," "When Life is a Failure," and "How to Live the Life Spiritual." We invite correspondence from those who hold varying points of view, and have made arrangements to devote ample space to the ventilation of the questions raised. By this means it is hoped to arouse the widest possible interest and to help prepare the way for the great movement of awakening throughout the Churches, the professions, and the people of all classes, which should tend materially to raise the standard of life throughout the country. Everything that can help to make the British race worthier of the higher responsibilities which must devolve upon the British nation and Empire in increasing volume as the war proceeds towards peace and after its declaration is to be encouraged and furthered by the Press of all sections. We hope that each section will devote space and sustained efforts to further the awakening movement now in preparation.

